# Cluster-Delay Mean Square Consensus of Stochastic Multi-Agent Systems with Impulse Time Windows

**DOI:** 10.3390/e23081033

**Published:** 2021-08-11

**Authors:** Huan Luo, Yinhe Wang, Ruidian Zhan, Xuexi Zhang, Haoxiang Wen, Senquan Yang

**Affiliations:** 1 School of Automation, Guangdong University of Technology, Guangzhou 510006, China; luohuan@sgu.edu.cn (H.L.); yinhewang@gdut.edu.cn (Y.W.); zxxnet@gdut.edu.cn (X.Z.); 2 School of Advanced Manufacturing, Guangdong University of Technology, Guangzhou 510006, China; 3 School of Intelligent Engineering, Shaoguan University, Shaoguan 512026, China; xiangxiang_0@163.com (H.W.); sqyangg@sgu.edu.cn (S.Y.); 4 Chipeye Microelectronics Foshan Ltd., Foshan 528225, China

**Keywords:** cluster-delay mean square consensus, multi-agent systems, stochastic disturbances, impulse time windows, impulsive control

## Abstract

This paper investigates the cluster-delay mean square consensus problem of a class of first-order nonlinear stochastic multi-agent systems with impulse time windows. Specifically, on the one hand, we have applied a discrete control mechanism (i.e., impulsive control) into the system instead of a continuous one, which has the advantages of low control cost, high convergence speed; on the other hand, we considered the existence of impulse time windows when modeling the system, that is, a single impulse appears randomly within a time window rather than an ideal fixed position. In addition, this paper also considers the influence of stochastic disturbances caused by fluctuations in the external environment. Then, based on algebraic graph theory and Lyapunov stability theory, some sufficiency conditions that the system must meet to reach the consensus state are given. Finally, we designed a simulation example to verify the feasibility of the obtained results.

## 1. Introduction

In today’s era, automation and intelligence are the mainstream directions of technological development. As a typical representative among them, multi-agent systems (MASs) [1] are widely used in epidemiology [2,3], sociology [4,5], engineering circles [6,7,8], and other fields with their powerful distributed integration capabilities. In [9,10], a concept called Holonic MAS was proposed, and subsequent researchers have achieved a series of meaningful results on this basis. As a key subject in the field of distributed collaborative control, the research on the consensus of MASs has also received increasingly more attention from the academic community, including group or cluster consensus [11,12,13], leader-following consensus [14,15,16], $H_{\infty }$ consensus [17,18,19], finite-time or fixed-time consensus [20,21,22], etc. In practical applications, MASs are required to simultaneously tend to multiple consensus states according to different task requirements. Specifically, MASs is divided into multiple clusters (i.e., subgroups) based on the degree of association between agents, and the states of all individuals included in each cluster eventually tend to be the same.

In particular, if a virtual state is selected as the consensus state of a certain cluster, and the remaining clusters’ consensus states are different delay states corresponding to the virtual state, such a case is called cluster-delay consensus, and it is also a special case of the group consensus. In [23], for a class first-order nonlinear MASs, the authors proposed the cluster-delay consensus problem for the first time and studied it through a continuity control strategy. Furthermore, in [24], a new type of pinning consensus protocol with intermittent effect was designed to ensure that the system can achieve the cluster-delay consensus. Moreover, by using the pinning leader-following approach, the cluster-delay consensus of first-order nonlinear MASs with aperiodic intermittent communication was studied in [25]. On the basis of these research work, the cluster-delay consensus problem with intermittent effects and layered intermittent communication was studied in [26] through tracking approach. In [27], the authors extend the research work on the first-order integrator system to more complex second-order system, and investigated the cluster-delay consensus problem of a class of second-order nonlinear MASs.

However, the above-mentioned works are all based on the continuity control protocol, which requires the agent to maintain continuous communication with its neighbors. First, it has higher requirements for communication guarantee capability. Second, it also increases the control cost. In applications, the agent may not be able to obtain the neighbor’s information continuously, and the above research results will no longer be applicable. At this time, it is conservative. Different from the traditional continuous control method, impulsive control has the advantages of low control cost, high control efficiency, and strong adaptability. Consequently, it is widely used in the research on leader-following consensus or group consensus of MASs [28,29,30]. Therefore, it is necessary to study the cluster-delay consensus of MASs via impulsive control [31]. In addition, there are some other interesting control mechanisms, such as the fuzzy control-based on sampled data [32,33], which is widely used in the consensus or synchronization problems research of MASs. Actually, the impulsive controller may not accurately act on the system at an ideal fixed impulse instant, it may be earlier or later. Therefore, the impulse appears randomly within a time window that is defined as an impulse time window in [34], and the window must be known. In order to obtain more general results, it is undoubtedly necessary to introduce the concept of impulse time window into the study of cluster-delay consensus. In general, MASs is also affected by stochastic disturbances caused by fluctuations in the external environment. Therefore, it is also necessary to study the cluster-delay consensus of nonlinear stochastic MASs (SMASs) [35].

Inspired by the above discussion, based on impulsive control strategy, we study the cluster-delay consensus of a class of SMASs with impulse time windows. The main contributions are as follows.

- In this paper, the cluster-delay consensus problem of MASs is studied based on the concept of the impulse time window for the first time. From this perspective, our contribution is mainly reflected in solving the problem of how to reasonably preset the impulsive time sequence under the new application background. In other words, setting the corresponding impulse time window layout according to our research results can ensure that MASs achieve cluster-delay consensus under the action of non-fixed position impulsive control signals.
- This paper studies the cluster-delay mean square consensus problem of MASs based on the uncertainty model for the first time, and gives a sufficient mean square consensus criterion through the It$\hat{o}$ formula, which deepens and expands the current research jobs to a certain extent.

The organization of the rest of this paper is shown below. Section 2 introduces the commonly used symbols and the content of algebraic graph theory. In Section 3, the research problem is described and the corresponding system model is constructed. In Section 4, the corresponding consensus criterion is derived through the analysis method. Then, numerical simulation is given in Section 5 to verify the validity of the obtained results. Section 6 summarizes the work of the full text.

## 2. Notation and Preliminaries

The symbols R, $R^{m\times n}$, and N denote the sets of real numbers, m×n matrices, and natural numbers, respectively. $R^{n}$ denotes *n*-dimensional Euclidean space. $N^{+}$ denotes the set of positive integers. Symbols |x| and ∥x∥ represent the absolute value and the Euclidean norm for x∈R and $x\in R^{n}$, respectively. The Kronecker product and the Kolmogorov operator are denoted by ⊗ and L, respectively. For $\varrho \in R^{m\times n}$, ${\left(\varrho \right)}^{T}$ and $\lambda_{max}\left(\varrho \right)$ denote the transpose and the maximal eigenvalue of the matrix ϱ, respectively. E(·) denotes the mathematic expectation of corresponding variable. Let w(t) be the Wiener process with *m*-dimensional, which defined on the complete probability space $\left(\Omega ,F,{\left\{F_{t}\right\}}_{t\geq 0},P\right)$ with filtration ${\left\{F_{t}\right\}}_{t\geq 0}$. diag(·) represents a diagonal matrix.

Consider a class of MASs of *N* agents, and the system’s communication topology can be denoted by digraph G=(D,E,A) without self-circulation, where $D=\left\{D_{1},\ldots ,D_{N}\right\}$ is the set of nodes, $E=\{\left(D_{j},D_{i}\right):i,j=1,\ldots ,N\}\subset D\times D$ is the set of edges, $A=[a_{ij}]$ is the weighted adjacency matrix with order N×N. If $D_{i}$ receives the state information of $D_{j}$, the weight of edge $\left(D_{j},D_{i}\right)$ is greater than 0, for convenience, let $a_{ij}=1$. Otherwise, $a_{ij}=0$. The degree matrix is denoted by $D=\mathrm{diag}(d_{i},i=1,\ldots ,N)$, where $d_{i}=\sum_{j=1,j\neq i}^{N}a_{ij}$. Then, $L=D-A=[l_{ij}]$ denotes the Laplacian matrix, where $l_{ij}=\left\{\begin{array}{c} -a_{ij},i\neq j\\ -\sum_{j=1,j\neq i}^{N}l_{ij},i=j \end{array}\right.$. If MASs contains a leader $D_{0}$, then the connection matrix is denoted by $C=\mathrm{diag}(c_{1},\ldots ,c_{N})$. When agent *i* receives the leader’s information, for convenience, let the weight of edge $\left(D_{0},D_{i}\right)$ be $c_{i}=1$. Otherwise, $c_{i}=0$. If all agents can receive the leader’s information, the leader is called a globally reachable node (i.e., C is an N-dimensional identity matrix).

Similar to the work in [23], we give the explanation and description of the following concepts in advance to facilitate the understanding of the cluster-delay consensus. If MASs is divided into multiple clusters labeled by ${\hat{D}}_{1}$, *…*, ${\hat{D}}_{Q}$, respectively, and let the index sets of *Q* clusters be ${\hat{D}}_{1}=\left\{1,2,\cdots ,m_{1}\right\}$, *…*, ${\hat{D}}_{\bar{i}}=\left\{m_{1}+m_{2}+\cdots +m_{\bar{i}-1}+1,\ldots ,m_{1}+\cdots +m_{\bar{i}-1}+m_{\bar{i}}\right\}$, *…*, ${\hat{D}}_{Q}=\left\{m_{1}+m_{2}+\cdots +m_{Q-1}+1,\ldots ,N\right\}$, where $N=m_{1}+\cdots +m_{Q}$, $\bar{i}\in \left\{1,2,\cdots ,Q\right\}$, $Q\in N^{+}$, $m_{\bar{i}}\in N^{+}$. If the *i*-th agent belongs to a certain cluster, let the subscript of the index set of the cluster be $\hat{i}$, that is, $i\in {\hat{D}}_{\hat{i}}$ and $\hat{i}=1,\ldots ,Q$. As for why these concepts are introduced, we will describe them in detail in the following part.

## 3. Problem Description and Model Construction

We consider a first-order nonlinear SMASs composed of N agents, the *i*-th agent’s dynamic is defined by

(1) $$\begin{array}{cc} dx_{i}\left(t\right)= & \left[f\left(t,x_{i}\left(t\right)\right)+Ax_{i}\left(t\right)-\rho_{\hat{i}}(S_{\hat{i}}\left(t\right)-S_{1}(t-\tau_{\hat{i}})\right)\\ & +u_{i}\left(t\right)]dt+\xi \left(t,x_{i}\left(t\right)\right)dw\left(t\right), \end{array}$$

where $x_{i}\left(t\right)\in R^{n}$ is the state vector (or displacement state vector in some physical systems), A is a known constant matrix, $f:R\times R^{n}\to R^{n}$ is a continuous nonlinear function, $u_{i}\left(t\right)\in R^{n}$ is the control input, $S_{\hat{i}}\left(t\right)\in R^{n}$ is the state vector of the virtual leader of the cluster where the *i*-th agent belongs, $S_{1}(t-\tau_{\hat{i}})$ is the delay state of the virtual leader of the first cluster, $\tau_{\hat{i}}$ is the time delay, and $\tau_{1}=0$, $\rho_{\hat{i}}$ is the coupling strength, $\xi :R\times R^{n}\to R^{n\times m}$ stands for the noisy intensity function. Besides, w(t) is an m-dimensional Wiener process defined on the complete probability space $\left(\Omega ,F,{\left\{F_{t}\right\}}_{t\geq 0},P\right)$ with filtration ${\left\{F_{t}\right\}}_{t\geq 0}$ which satisfies the usual conditions (i.e., $F_{0}$ contains all *P*-null sets and $F_{t}$ is right continuous), and $w_{i}\left(t\right)$ and $w_{j}\left(t\right)$ are independent of each other when i≠j.

**Assumption** **1.** *Each agent has a communication connection with the virtual leader of the cluster to which it belongs, and the first cluster’s virtual leader has a communication connection with the virtual leaders of all other clusters.*

Different from continuous control strategy, the following impulsive controller is designed.

(2) $$\begin{array}{cc} u_{i}\left(t\right)= & \sum_{k=1}^{+\infty }\delta \left(t-t_{k}\right)(K(\alpha \sum_{j=1}^{N}a_{ij}(x_{j}\left(t\right)-S_{\hat{j}}\left(t\right)\\ & -\left(x_{i}\left(t\right)-S_{\hat{i}}\left(t\right)\right))-\beta \left(x_{i}\left(t\right)-S_{\hat{i}}\left(t\right)))\right), \end{array}$$

where δ(t) is the Dirac function, *K* is an impulsive gain matrix, α∈(0,1) and β∈(0,1) are the coupling strengths, $\left\{t_{k}\right\}$ satisfies $0\leq t_{0}<\cdots <t_{k}$ and $\lim_{k\to +\infty }t_{k}=+\infty$, $x_{i}\left(t\right)$ is right continuous at each $t_{k}$, i.e., $\lim_{h\to 0^{+}}x_{i}\left(t_{k}+h\right)=x_{i}\left(t_{k}\right)$, $k\in N^{+}$.

**Remark** **1.** *In [23], the continuity control protocol was designed as $u_{i}\left(t\right)=-k_{\hat{i}}\left(s_{i}\left(t\right)-s_{1}\left(t-\tau_{\hat{i}}\right)\right)-\sigma_{i}\left(x_{i}\left(t\right)-s_{\hat{i}}\left(t\right)\right)+\sum_{j\notin V_{\hat{i}}}l_{ij}\left(x_{j}\left(t\right)-x_{i}\left(t\right)\right)$. It is easy to see that the i-th agent needs to continuously obtain the state information of its neighbors j to update the control signal so that the control cost, as well as the communication burden, are higher. In other words, once the communication between agents cannot be maintained continuously, the above-mentioned controller will lose its effectiveness. However, the impulsive controller is shown in (2) only acts on the system at a series of discrete-time points, which reduces the control cost and the communication volume effectively in the control process. Therefore, the impulsive control mechanism is suitable for some actual environments with a limited communication load, and its adaptability is stronger. In addition, when the state error between the agent and its leader is large, the agent’s state will have a large instantaneous jump via the impulsive control, so the response speed is faster than that in other methods.*

**Figure 1:**
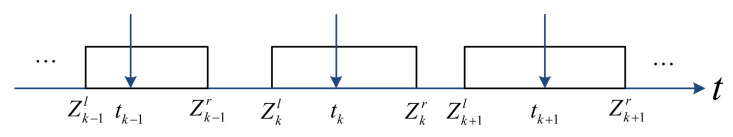
Impulse time windows on the time axis.

For the impulsive control mechanism, we need to preset an impulsive time sequence and assume that the impulse acts on the system at these given ideal moments. However, due to the limitations of physical equipment and objective environments, in practical applications, the real instant of impulse appearance is earlier or later than the ideal moment. In [34], the authors proposed a concept called impulse time window to describe this common phenomenon, as shown in Figure 1, where $Z_{k}^{l}$ and $Z_{k}^{r}$ are the left and right end points of the *k*-th window, respectively, $t_{k}$ is the real impulsive control moment, $t_{0}\leq Z_{1}^{l}<t_{1}<Z_{1}^{r}<Z_{2}^{l}<\cdots$. It can be seen from Figure 1 that impulse appears randomly in the window, and each window corresponds to only one impulse.

We introduce the corresponding virtual leaders into each cluster of SMASs, and their dynamic equations are described by

(3) $$\begin{array}{c} dS_{y}\left(t\right)=\left[f\left(t,S_{y}\left(t\right)\right)+AS_{y}\left(t\right)-\rho_{y}\left(S_{y}\left(t\right)-S_{1}\left(t-\tau_{y}\right)\right)\right]dt+\xi \left(t,S_{y}\left(t\right)\right)dw\left(t\right), \end{array}$$

where y=1,…,Q.

**Figure 2:**
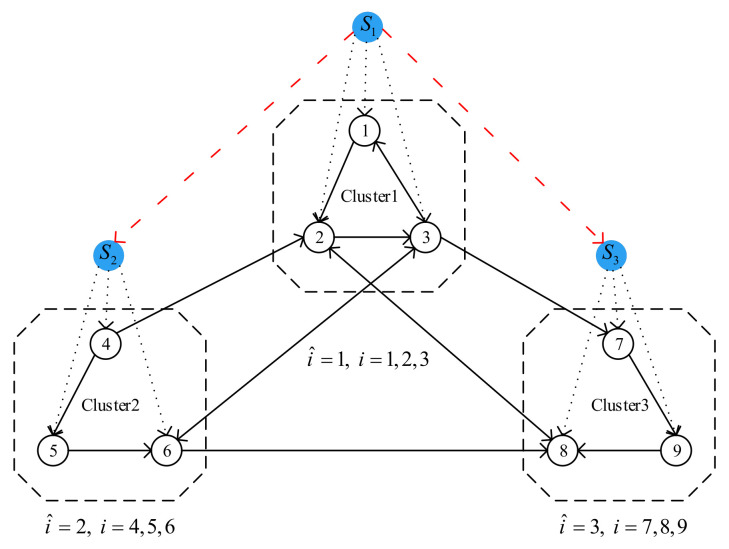
Multi-agent systems with virtual leaders.

**Remark** **2.** *Because our research object is a SMASs without real leaders, in order to facilitate group control, we assign corresponding virtual leaders to each cluster in the system. Note that to make it easier to construct an error system, the virtual leader and the follower agent have the same dynamics. As the follower agents in each cluster need to reach their respective consensus states, the number of virtual leaders is the same as the number of clusters in the system. At the same time, suppose there is a coupling relationship of state information between some virtual leaders, as shown in Figure 2.*

Let $e_{y}\left(t\right)=S_{y}\left(t\right)-S_{1}\left(t-\tau_{y}\right)$, $e\left(t\right)={\left(e_{1}^{T}\left(t\right),\cdots ,e_{Q}^{T}\left(t\right)\right)}^{T}$, $F\left(t,e_{y}\left(t\right)\right)=f\left(t,S_{y}\left(t\right)\right)-f\left(t-\tau_{y},S_{1}\left(t-\tau_{y}\right)\right)$, $\bar{F}\left(t,e\left(t\right)\right)={\left(F^{T}\left(t,e_{1}\left(t\right)\right),\cdots ,F^{T}\left(t,e_{Q}\left(t\right)\right)\right)}^{T}$, $\tilde{\xi }\left(t,e_{y}\left(t\right)\right)=\xi \left(t,S_{y}\left(t\right)\right)-\xi \left(t-\tau_{y},S_{1}\left(t-\tau_{y}\right)\right)$, $\bar{\xi }\left(t,e\left(t\right)\right)={\left({\tilde{\xi }}^{T}\left(t,e_{1}\left(t\right)\right),\cdots ,{\tilde{\xi }}^{T}\left(t,e_{Q}\left(t\right)\right)\right)}^{T}$. Then, according to (3), we can get the following error system.

(4) $$\begin{array}{c} de\left(t\right)=\left[\left(I_{Q}⊗I_{n}\right)\bar{F}\left(t,e\left(t\right)\right)+\left(I_{Q}⊗A\right)e\left(t\right)-\left(\Lambda ⊗I_{n}\right)e\left(t\right)\right]dt+\bar{\xi }\left(t,e\left(t\right)\right)dw\left(t\right), \end{array}$$

where $\Lambda =\mathrm{diag}\left(\rho_{1},\rho_{2},\cdots ,\rho_{Q}\right)$.

Next, based on (1) and (2), we have the system model with impulse time windows as follows.

(5) $$\left\{\begin{array}{cc} dx_{i}\left(t\right)= & \left[f\left(t,x_{i}\left(t\right)\right)+Ax_{i}\left(t\right)-\rho_{\hat{i}}\left(S_{\hat{i}}\left(t\right)-S_{1}\left(t-\tau_{\hat{i}}\right)\right)\right]dt+\xi \left(t,x_{i}\left(t\right)\right)dw\left(t\right),t\in \left[t_{0},Z_{1}^{l}\right]\\ & \cup \left[Z_{k}^{l},t_{k}\right)\cup \left(t_{k},Z_{k}^{r}\right],\\ \Delta x_{i}\left(t\right)= & x_{i}\left(t\right)-x_{i}\left(t^{-}\right)\\ = & K\left(\alpha \sum_{j=1}^{N}a_{ij}\left(x_{j}\left(t^{-}\right)-S_{\hat{j}}\left(t^{-}\right)-\left(x_{i}\left(t^{-}\right)-S_{\hat{i}}\left(t^{-}\right)\right)\right)-\beta \left(x_{i}\left(t^{-}\right)-S_{\hat{i}}\left(t^{-}\right)\right)\right),t=t_{k}. \end{array}\right.$$

Let ${\hat{x}}_{i}\left(t\right)=x_{i}\left(t\right)-S_{\hat{i}}\left(t\right)$, $\overset{˘}{f}\left(t,{\hat{x}}_{i}\left(t\right)\right)=f\left(t,x_{i}\left(t\right)\right)-f(t,S_{\hat{i}}\left(t\right))$, $\overset{˘}{\xi }\left(t,{\hat{x}}_{i}\left(t\right)\right)=\xi \left(t,x_{i}\left(t\right)\right)-\xi (t,S_{\hat{i}}\left(t\right))$. Then, error system (6) can be obtained as

(6) $$\left\{\begin{array}{cc} d{\hat{x}}_{i}\left(t\right)= & [\overset{˘}{f}\left(t,{\hat{x}}_{i}\left(t\right)\right)+A{\hat{x}}_{i}\left(t\right)]dt+\overset{˘}{\xi }\left(t,{\hat{x}}_{i}\left(t\right)\right)dw\left(t\right),t\in \left[t_{0},Z_{1}^{l}\right]\cup \left[Z_{k}^{l},t_{k}\right)\cup \left(t_{k},Z_{k}^{r}\right],\\ \Delta {\hat{x}}_{i}\left(t\right)= & {\hat{x}}_{i}\left(t\right)-{\hat{x}}_{i}\left(t^{-}\right)\\ = & K\left(\alpha \sum_{j=1}^{N}a_{ij}\left({\hat{x}}_{j}\left(t^{-}\right)-{\hat{x}}_{i}\left(t^{-}\right)\right)-\beta {\hat{x}}_{i}\left(t^{-}\right)\right),t=t_{k}. \end{array}\right.$$

Let $\hat{x}\left(t\right)={\left({\hat{x}}_{1}^{T}\left(t\right),\ldots ,{\hat{x}}_{N}^{T}\left(t\right)\right)}^{T}$, $\overset{˘}{F}\left(t,\hat{x}\left(t\right)\right)={\left({\overset{˘}{f}}^{T}\left(t,{\hat{x}}_{1}\left(t\right)\right),\cdots ,{\overset{˘}{f}}^{T}\left(t,{\hat{x}}_{N}\left(t\right)\right)\right)}^{T}$, $\hat{\xi }\left(t,\hat{x}\left(t\right)\right)={\left({\overset{˘}{\xi }}^{T}\left(t,{\hat{x}}_{1}\left(t\right)\right),\cdots ,{\overset{˘}{\xi }}^{T}\left(t,{\hat{x}}_{N}\left(t\right)\right)\right)}^{T}$. Therefore, system (6) can be rewritten as

(7) $$\left\{\begin{array}{cc} d\hat{x}\left(t\right)= & \left[\left(I_{N}⊗A\right)\hat{x}\left(t\right)+\left(I_{N}⊗I_{n}\right)\overset{˘}{F}\left(t,\hat{x}\left(t\right)\right)\right]dt+\hat{\xi }\left(t,\hat{x}\left(t\right)\right)dw\left(t\right),t\in \left[t_{0},Z_{1}^{l}\right]\cup \left[Z_{k}^{l},t_{k}\right)\\ & \cup (t_{k},Z_{k}^{r}],\\ \hat{x}\left(t\right)= & \Omega \hat{x}\left(t^{-}\right),t=t_{k}, \end{array}\right.$$

where $\Omega =I_{Nn}-\left(\beta I_{N}+\alpha L\right)⊗K$, $I_{N}$ and $I_{Nn}$ are the identity matrices with *N*-order and Nn-order, respectively.

**Remark** **3.** *In [23,24,25,26], the authors have adopted continuity control strategies to study the cluster-delay consensus problem of deterministic MASs. Obviously, this control method will greatly increase control costs and risks [36]. In contrast, this paper is characterized in that the influence of stochastic disturbances is considered, and what is more, it adopts a more advantageous impulsive control strategy. Therefore, the results obtained in this paper are suitable for actual scenarios in the presence of stochastic disturbances and limited communication load. Compared with the work in [31], the system model researched in this paper is more complicated, that is, the concepts of stochastic disturbances and impulse time window are introduced in the construction of the model and the controller, respectively. When the impulse signal appears jitter or drift, the obtained results effectively solve the problem of how to preset the impulse time sequence. In addition, compared with the research work related to the impulse time window, this paper studies the cluster-delay consensus problem of a class of nonlinear SMASs for the first time, and our work is mainly to explore the feasibility of combining these two different research fields. Although the authors considered the influence of random noises in [35,37], the continuity control strategy they applied may bring a great communication burden to the actual control. In this regard, by applying impulsive control mechanism, our paper avoids this problem well.*

For the subsequent consensus analysis, we give the following necessary lemma, assumption, and definitions.

**Lemma** **1** **([38]).** *For vectors $x,\hat{y}\in R^{n}$ and constant σ>0, we can get $x^{T}\hat{y}+{\hat{y}}^{T}x\leq \sigma x^{T}x+\sigma^{-1}{\hat{y}}^{T}\hat{y}$.*

**Assumption** **2.** *$\forall x_{i},x_{j}\in R^{n}$, there exist Lipschtiz constants ϕ and $\hat{\phi }$ such that $∥f\left(t,x_{i}\right)-f\left(t,x_{j}\right)∥\leq \phi ∥x_{i}-x_{j}∥$ and $∥\xi \left(t,x_{i}\right)-\xi \left(t,x_{j}\right)∥\leq \hat{\phi }∥x_{i}-x_{j}∥$.*

**Definition** **1** **([23]).** *The SMASs with (3) and (5) are said to reach cluster mean square consensus, if there exist the solutions of (3) and (5) such that $\lim_{t\to +\infty }E(∥{\hat{x}}_{i}\left(t\right){∥}^{2})=0$, where ${\hat{x}}_{i}\left(t\right)=x_{i}\left(t\right)-S_{\hat{i}}\left(t\right)$.*

**Definition** **2** **([23]).** *The SMASs with (3) are said to reach delay mean square consensus, if there exist the solutions of (3) such that $\lim_{t\to +\infty }E(∥e_{y}\left(t\right){∥}^{2})=0$, where $e_{y}\left(t\right)=S_{y}\left(t\right)-S_{1}\left(t-\tau_{y}\right)$.*

**Definition** **3.** *The SMASs with (3) and (5) are said to reach cluster-delay mean square consensus, if there exist the solutions of (3) and (5) such that $\lim_{t\to +\infty }E(∥{\hat{x}}_{i}\left(t\right){∥}^{2})=0$, and $\lim_{t\to +\infty }E(∥e_{y}\left(t\right){∥}^{2})=0$, where ${\hat{x}}_{i}\left(t\right)=x_{i}\left(t\right)-S_{\hat{i}}\left(t\right)$ and $e_{y}\left(t\right)=S_{y}\left(t\right)-S_{1}\left(t-\tau_{y}\right)$.*

**Remark** **4.** *As mentioned above, for ease of understanding, we have provided three different definitions of consensus. Obviously, only when the given conditions in Definitions 1 and 2 are met at the same time, Definition 3 related to cluster-delay consensus needed in this paper can be established. In other words, Definition 3 includes Definitions 1 and 2, and Definitions 1 and 2 are independent of each other. This also facilitates the step-by-step proof of the following consensus analysis part.*

## 4. Consensus Analysis

In this section, based on the Lyapunov stability theory and combined with the It$\hat{o}$ formula, we conduct a theoretical analysis of the cluster-delay consensus problem of the uncertain MASs and give the corresponding consensus criterion. The core idea of the proof is to transform the consensus problem of the original system into the stability analysis problem of the error system. According to Definition 3, the work of this part needs to be divided into two parts, namely, the proof of cluster mean square consensus and delay mean square consensus.

**Theorem** **1.** *Under Assumptions 1–2, for the involved scalars σ>0, ϕ>0, and $\hat{\phi }>0$ satisfying the following conditions (1)–(2), if there exist the solutions of (3) and (5) such that $\lim_{t\to +\infty }E(∥{\hat{x}}_{i}\left(t\right){∥}^{2})=0$, and $\lim_{t\to +\infty }E(∥e_{y}\left(t\right){∥}^{2})=0$, then the SMASs with (3) and (5) will achieve cluster-delay mean square consensus.*

(1) There exists a constant ϑ>1 such that $\ln\left(\vartheta \lambda^{*}\right)+\hat{\rho }\left(Z_{k+1}^{l}-Z_{k}^{l}\right)\;\leq \;0$, where $\lambda^{*}=\lambda_{\max}\left(\Omega^{T}\Omega \right)$, $\Omega =I_{Nn}-\left(\beta I_{N}+\alpha L\right)⊗K$, $\hat{\rho }=\gamma +\sigma +\sigma^{-1}\phi^{2}+{\hat{\phi }}^{2}$, and $\gamma =\lambda_{\max}\left(I_{N}⊗\left(A+A^{T}\right)\right)$.

*(2) There exists a negative definite matrix $I_{Q}⊗A-\Lambda ⊗I_{n}$ such that $\tilde{\rho }=\sigma +\sigma^{-1}\phi^{2}+{\hat{\phi }}^{2}+2\lambda_{\max}\left(I_{Q}⊗A-\Lambda ⊗I_{n}\right)<0$, where $\Lambda =\mathrm{diag}\left(\rho_{1},\rho_{2},\cdots ,\rho_{Q}\right)$.*

Proof. *(a): cluster mean square consensus*

Construct the following Lyapunov function:

(8) $$V\left(t,\hat{x}\left(t\right)\right)={\hat{x}}^{T}\left(t\right)\hat{x}\left(t\right).$$

The stochastic derivative of (8) is derived by the $It\hat{o}$ formula along the trajectory of system (7) as follows.

(9) $$dV\left(t,\hat{x}\left(t\right)\right)=LV\left(t,\hat{x}\left(t\right)\right)+2{\hat{x}}^{T}\left(t\right)\hat{\xi }\left(t,\hat{x}\left(t\right)\right)dw\left(t\right),$$

(10) $$\begin{array}{cc} LV(t,\hat{x}\left(t\right))= & 2{\hat{x}}^{T}\left(t\right)\left[\left(I_{N}⊗A\right)\hat{x}\left(t\right)+\left(I_{N}⊗I_{n}\right)\overset{˘}{F}\left(t,\hat{x}\left(t\right)\right)\right]+\mathrm{trace}\left[{\hat{\xi }}^{T}\left(t,\hat{x}\left(t\right)\right)\hat{\xi }\left(t,\hat{x}\left(t\right)\right)\right]. \end{array}$$

According to Assumption 2 and Lemma 1, from (10), we have

(11) $$2{\hat{x}}^{T}\left(t\right)\left(I_{N}⊗A\right)\hat{x}\left(t\right)\leq \gamma V\left(t,\hat{x}\left(t\right)\right),$$

(12) $$\begin{array}{c} 2{\hat{x}}^{T}\left(t\right)\left(I_{N}⊗I_{n}\right)\overset{˘}{F}\left(t,\hat{x}\left(t\right)\right)\\ \leq \sigma {\hat{x}}^{T}\left(t\right)\hat{x}\left(t\right)+\sigma^{-1}{\overset{˘}{F}}^{T}\left(t,\hat{x}\left(t\right)\right)\overset{˘}{F}\left(t,\hat{x}\left(t\right)\right)\\ \leq \left(\sigma +\sigma^{-1}\phi^{2}\right)V\left(t,\hat{x}\left(t\right)\right) \end{array}$$

and

(13) $$\mathrm{trace}\left[{\hat{\xi }}^{T}\left(t,\hat{x}\left(t\right)\right)\hat{\xi }\left(t,\hat{x}\left(t\right)\right)\right]\leq {\hat{\phi }}^{2}V\left(t,\hat{x}\left(t\right)\right).$$

For $t\in [t_{k-1},t_{k})$, assume that Δt is a small enough positive constant such that $t+\Delta t\in (t_{k-1},t_{k})$, then one has

(14) $$\begin{array}{cc} \; & EV\left(t+\Delta t,\hat{x}\left(t+\Delta t\right)\right)-EV\left(t,\hat{x}\left(t\right)\right)=\int_{t}^{t+\Delta t}ELV\left(s,\hat{x}\left(s\right)\right)ds. \end{array}$$

By (11)–(14), we can obtain

(15) $$D^{+}EV\left(t,\hat{x}\left(t\right)\right)=ELV\left(t,\hat{x}\left(t\right)\right)\leq \hat{\rho }EV\left(t,\hat{x}\left(t\right)\right).$$

When $t\in \left[t_{0},Z_{1}^{l}\right)$ and $t\in \left[Z_{k}^{r},Z_{k+1}^{l}\right)$, from (15), we have

(16) $$EV\left(Z_{1}^{l},\hat{x}\left(Z_{1}^{l}\right)\right)\leq EV\left(t_{0},\hat{x}\left(t_{0}\right)\right)\exp\left(\hat{\rho }\left(Z_{1}^{l}-t_{0}\right)\right),$$

(17) $$EV\left(Z_{k+1}^{l},\hat{x}\left(Z_{k+1}^{l}\right)\right)\leq EV\left(Z_{k}^{r},\hat{x}\left(Z_{k}^{r}\right)\right)\exp\left(\hat{\rho }\left(Z_{k+1}^{l}-Z_{k}^{r}\right)\right).$$

Let k=1. For $t\in \left[Z_{1}^{l},t_{1}\right)$, it holds that

(18) $$EV\left(t_{1}^{-},\hat{x}\left(t_{1}^{-}\right)\right)\leq EV\left(t_{0},\hat{x}\left(t_{0}\right)\right)\exp\left(\hat{\rho }\left(t_{1}-t_{0}\right)\right).$$

When $t=t_{k}$, one has

(19) $$EV\left(t_{k}\right)=E\left({\hat{x}}^{T}\left(t_{k}^{-}\right)\Omega^{T}\Omega \hat{x}\left(t_{k}^{-}\right)\right)\leq \lambda^{*}EV\left(t_{k}^{-},\hat{x}\left(t_{k}^{-}\right)\right).$$

Thus, from (19), we have

(20) $$EV\left(t_{1},\hat{x}\left(t_{1}\right)\right)\leq \lambda^{*}EV\left(t_{1}^{-},\hat{x}\left(t_{1}^{-}\right)\right).$$

For $t\in \left(t_{1},Z_{1}^{r}\right)$, we can get

(21) $$\begin{array}{cc} \; & EV\left(Z_{1}^{r},\hat{x}\left(Z_{1}^{r}\right)\right.)\\ & \leq EV\left(t_{1},\hat{x}\left(t_{1}\right)\right)\exp\left(\hat{\rho }\left(Z_{1}^{r}-t_{1}\right)\right)\\ \; & \leq \lambda^{*}EV\left(t_{1}^{-},\hat{x}\left(t_{1}^{-}\right)\right)\exp\left(\hat{\rho }\left(Z_{1}^{r}-t_{1}\right)\right)\\ \; & \leq \lambda^{*}EV\left(t_{0},\hat{x}\left(t_{0}\right)\right)\exp\left(\hat{\rho }\left(Z_{1}^{r}-t_{0}\right)\right). \end{array}$$

When $t\in \left[Z_{1}^{r},Z_{2}^{l}\right)$, by (17) and (21), it follows that

(22) $$\begin{array}{cc} EV\left(Z_{2}^{l},\hat{x}\left(Z_{2}^{l}\right)\right) & \leq EV\left(Z_{1}^{r},\hat{x}\left(Z_{1}^{r}\right)\right)\exp\left(\hat{\rho }\left(Z_{2}^{l}-Z_{1}^{r}\right)\right)\\ \; & \leq \lambda^{*}EV\left(t_{0},\hat{x}\left(t_{0}\right)\right)\exp\left(\hat{\rho }\left(Z_{2}^{l}-t_{0}\right)\right). \end{array}$$

Let k=2. When $t\in \left[Z_{2}^{r},Z_{3}^{l}\right)$, it yields

$$EV\left(Z_{3}^{l},\hat{x}\left(Z_{3}^{l}\right)\right)\leq {\left(\lambda^{*}\right)}^{2}EV\left(t_{0},\hat{x}\left(t_{0}\right)\right)\exp\left(\hat{\rho }\left(Z_{3}^{l}-t_{0}\right)\right).$$

By analogy, for $t\in \left[Z_{k}^{l},Z_{k+1}^{l}\right)$, if there exists a constant ϑ>1 such that $\ln\left(\vartheta \lambda^{*}\right)+\hat{\rho }\left(Z_{k+1}^{l}-Z_{k}^{l}\right)\leq 0$, then we have

(23) $$\begin{array}{cc} EV(t,\hat{x}\left(t\right)) & \leq {\left(\lambda^{*}\right)}^{k}EV\left(t_{0},\hat{x}\left(t_{0}\right)\right)\exp\left(\hat{\rho }\left(t-t_{0}\right)\right)\\ \; & \leq EV\left(t_{0},\hat{x}\left(t_{0}\right)\right)\exp\left(\hat{\rho }\left(t-Z_{k}^{l}\right)\right)\lambda^{*}\exp\left(\hat{\rho }\left(Z_{k}^{l}-Z_{k-1}^{l}\right)\right)\cdots \lambda^{*}\exp\left(\hat{\rho }\left(Z_{1}^{l}-t_{0}\right)\right)\\ \; & \leq \frac{1}{\vartheta^{k}}EV\left(t_{0},\hat{x}\left(t_{0}\right)\right)\exp\left(\hat{\rho }\left(t-Z_{k}^{l}\right)\right). \end{array}$$

From (23), it can be seen that $EV(t,\hat{x}\left(t\right))\to 0$ when t→∞. That is, $\lim_{t\to \infty }E(∥x_{i}\left(t\right)-S_{\hat{i}}\left(t\right){∥}^{2})=0$. Therefore, the SMASs with (3) and (5) can achieve the cluster mean square consensus.

*(b): delay mean square consensus*

Construct the following Lyapunov function:

(24) $$V\left(t,e\left(t\right)\right)=e^{T}\left(t\right)e\left(t\right).$$

The stochastic derivative of (24) is derived by the $It\hat{o}$ formula along the trajectory of system (4) as follows.

(25) $$dV\left(t,e\left(t\right)\right)=LV\left(t,e\left(t\right)\right)+2e^{T}\left(t\right)\bar{\xi }\left(t,e\left(t\right)\right)dw\left(t\right),$$

(26) $$\begin{array}{cc} LV(t,e(t))= & 2e^{T}\left(t\right)\left[\left(I_{Q}⊗A-\Lambda ⊗I_{n}\right)e\left(t\right)+\left(I_{Q}⊗I_{n}\right)\bar{F}\left(t,e\left(t\right)\right)\right]\\ & +\mathrm{trace}[{\bar{\xi }}^{T}\left(t,e\left(t\right)\right)\bar{\xi }\left(t,e\left(t\right)\right)]. \end{array}$$

Similar to (12) and (13), we have

(27) $$\begin{array}{c} 2e^{T}\left(t\right)\left(I_{Q}⊗I_{n}\right)\bar{F}\left(t,e\left(t\right)\right)\\ \leq \sigma e^{T}\left(t\right)e\left(t\right)+\sigma^{-1}{\bar{F}}^{T}\left(t,e\left(t\right)\right)\bar{F}\left(t,e\left(t\right)\right)\\ \leq \left(\sigma +\sigma^{-1}\phi^{2}\right)V\left(t,e\left(t\right)\right). \end{array}$$

and

(28) $$\mathrm{trace}\left[{\bar{\xi }}^{T}\left(t,e\left(t\right)\right)\bar{\xi }\left(t,e\left(t\right)\right)\right]\leq {\hat{\phi }}^{2}V\left(t,e\left(t\right)\right).$$

Furthermore, one has

(29) $$\begin{array}{c} 2e^{T}\left(t\right)\left(I_{Q}⊗A-\Lambda ⊗I_{n}\right)e\left(t\right)\leq 2\lambda_{\max}\left(I_{Q}⊗A-\Lambda ⊗I_{n}\right)V\left(t,e\left(t\right)\right). \end{array}$$

In the same way, we can get the following inequality similar to (14).

(30) $$\begin{array}{cc} \; & EV\left(t+\Delta t,e\left(t+\Delta t\right)\right)-EV\left(t,e\left(t\right)\right)=\int_{t}^{t+\Delta t}ELV\left(s,e\left(s\right)\right)ds. \end{array}$$

According to (27)–(30), we can obtain

(31) $$D^{+}EV\left(t,e\left(t\right)\right)=ELV\left(t,e\left(t\right)\right)\leq \tilde{\rho }EV\left(t,e\left(t\right)\right).$$

From (31), one has

(32) $$EV\left(t,e\left(t\right)\right)\leq EV\left(t_{0},e\left(t_{0}\right)\right)\exp\left(\tilde{\rho }\left(t-t_{0}\right)\right).$$

At this time, if matrix $I_{Q}⊗A-\Lambda ⊗I_{n}$ is negative definite and its maximum eigenvalue satisfying $\tilde{\rho }<0$, then it can be known from (32) that EV(t,e(t))→0 when t→∞. That is, $\lim_{t\to \infty }E(∥S_{y}\left(t\right)-S_{1}\left(t-\tau_{y}\right){∥}^{2})=0$. Consequently, the SMASs with (3) can reach the delay mean square consensus.

According to parts (a) and (b), we can say that the SMASs with (3) and (5) can reach the cluster-delay mean square consensus. This completes the proof. □

**Remark** **5.** By condition (1), we have $Z_{k+1}^{l}-Z_{k}^{l}\leq \frac{-\ln\left(\vartheta \lambda^{*}\right)}{\hat{\rho }}$, where parameters $\lambda^{*}<1$ and $\hat{\rho }$ can be obtained by simple calculations. Without loss of generality, ϑ can be equivalently regarded as an adjustable variable that satisfies $\vartheta \lambda^{*}<1$. Obviously, the artificial preset of the impulse time windows and the selection of the value of ϑ influence each other. When the interval between adjacent impulse time windows is designed to be larger, this means that ϑ needs to be larger to ensure that $\vartheta \lambda^{*}<1$ holds. At this time, it can be seen from (23) that the convergence speed of the error system will decrease. Reflected in the actual control, the impulsive interval may become larger due to the above-mentioned design changes, and the number of impulses within a certain period of time will be reduced, resulting in a slower system convergence speed, and vice versa.

**Remark** **6.** *We know that A is a known real matrix, and its value depends on the inherent dynamic behavior of SMASs. In other words, for a particular system, the value of A cannot be adjusted. Therefore, to satisfy condition (2), we can only adjust the diagonal matrix *Λ* composed of virtual coupling strengths $\rho_{1}$, ⋯, and $\rho_{Q}$. According to condition (2), we can see that the stronger the coupling strengths are, the easier the inequality $\tilde{\rho }<0$ is satisfied. At the same time, the delay mean-square consensus of SMASs may be realized faster.*

**Remark** **7.** *Different from the general literature, the proof method in this paper combines the characteristics of multiple current methods and has been successfully applied to the study of the SMASs’ cluster-delay mean square consensus problem. In a sense, this is an extension of current research methods. Moreover, how to construct the dynamic equation of the virtual leaders, how to design an impulsive controller and adjust its parameters, how to design a reasonable impulse time sequence, and how to design a reasonable simulation program to verify the effectiveness of the research method are challenging jobs. In addition, through the above research, we can reasonably preset the impulse time sequence to avoid the possible adverse effects of the digital signal’s jitter or drift on the system when the MASs are facing stochastic disturbances. In practical applications, the target MASs studied in this paper can be cluster drones flying in formation, numerous unmanned vehicles on the road, or a network of multiple power stations.*

## 5. Numerical Simulation

Next, we design a simulation example to verify the validity of the obtained results.

**Example** **1.** *Consider a first-order nonlinear SMASs composed of 9 agents, and its topology graph is shown in Figure 2. In order to easily identify the state trajectory of each agent in the simulation diagram, we choose a class of one-dimensional variable as the agent’s state, namely, n=1.*

Let the initial states $x_{1}\left(t_{0}\right)=1$, $x_{2}\left(t_{0}\right)=10$, $x_{3}\left(t_{0}\right)=-12$, $x_{4}\left(t_{0}\right)=-3$, $x_{5}\left(t_{0}\right)=-16$, $x_{6}\left(t_{0}\right)=5$, $x_{7}\left(t_{0}\right)=15$, $x_{8}\left(t_{0}\right)=-7$, $x_{9}\left(t_{0}\right)=9$, $S_{1}\left(t_{0}\right)=-1$, $S_{2}\left(t_{0}\right)=-6$, and $S_{3}\left(t_{0}\right)=3$. Let functions $f\left(t,x_{i}\left(t\right)\right)=x_{i}\left(t\right)\sin\left(\tan t\right)$, $f\left(S_{y}\left(t\right),t\right)=S_{y}\left(t\right)\sin\left(\tan t\right)$, and $\xi \left(t,x_{i}\left(t\right)\right)=0.16\left|\cos\left(t\right)\right|x_{i}\left(t\right)$. Obviously, we can choose Lipschtiz constants ϕ=1 and $\hat{\phi }=0.16$. Furthermore, let A=1, α=0.2, β=0.8, K=diag(0.3,⋯,0.3), $\rho_{2}=\rho_{3}=20$, $\tau_{2}=0.2$, and $\tau_{3}=0.3$. Based on the above parameters, we have $\lambda^{*}=0.6111$, γ=2. We can choose parameters ϑ=1.2 and σ=1. Then, it can be calculated according to condition 1) in Theorem 1 that $Z_{k+1}^{l}-Z_{k}^{l}\leq 0.103$. In addition, it is clear that the matrix $I_{Q}⊗A-\Lambda ⊗I_{n}$ is negative definite and satisfies the condition $\tilde{\rho }<0$.

**Figure 3:**
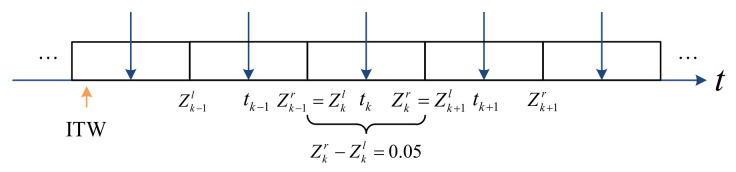
Design layout of impulse time window (ITW).

Finally, for convenience, we designed a class of layout of the impulse time windows as shown in Figure 3. Specifically, we stipulate that the width of each window in the figure is 0.05 and that the impulse appears in the center point of each window. In other words, $\forall k\in N^{+}$, we have $Z_{k+1}^{l}-Z_{k}^{l}=0.05$ and $t_{k+1}-t_{k}=0.05$.

**Figure 4:**
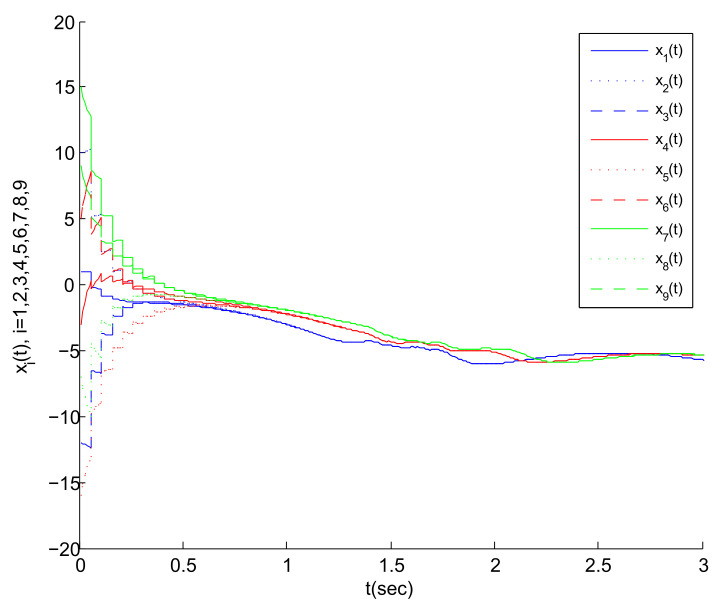
State trajectory of each agent under impulsive control.

**Figure 5:**
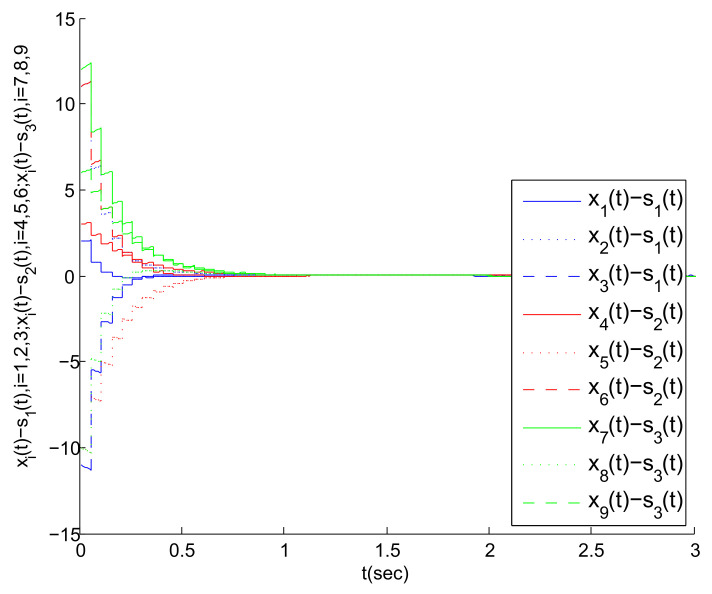
The state error trajectory for each agent in clusters.

**Figure 6:**
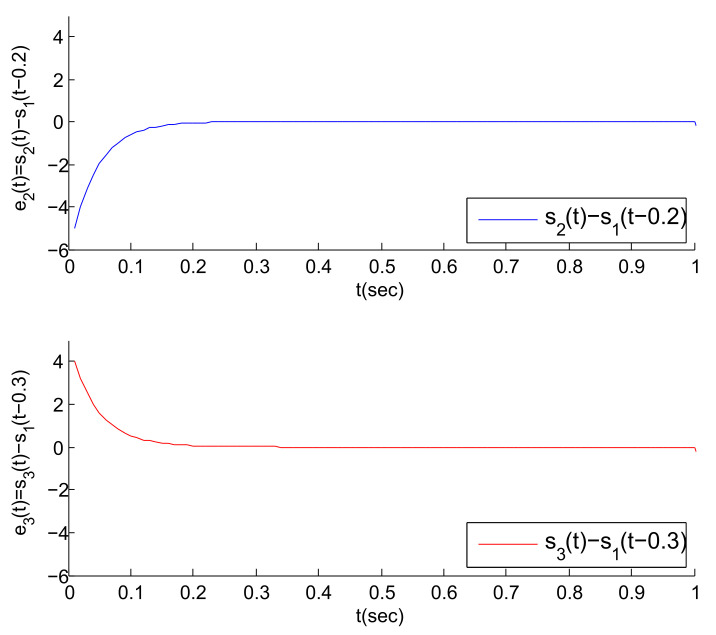
The error trajectories between $S_{2}\left(t\right)$, $S_{3}\left(t\right)$ and their virtual leaders’ states when $\rho_{2}=\rho_{3}=20$.

Based on the above work, Figure 4, Figure 5 and Figure 6 are obtained by Matlab platform as follows.

According to Figure 4, we can find that the MASs are divided into three clusters, and the states of the three agents in each cluster gradually tend to a common state (i.e., the virtual leader’s state). Correspondingly, the state error between each agent and its virtual leader also gradually tends to 0, as shown in Figure 5. Thus, the cluster mean square consensus of system (5) can be achieved. Obviously, it can be seen from Figure 6 that system (3) has achieved the delay mean square consensus. In summary, based on the impulse time windows, the cluster-delay mean square consensus of the SMASs with (3) and (5) can be realized.

**Figure 7:**
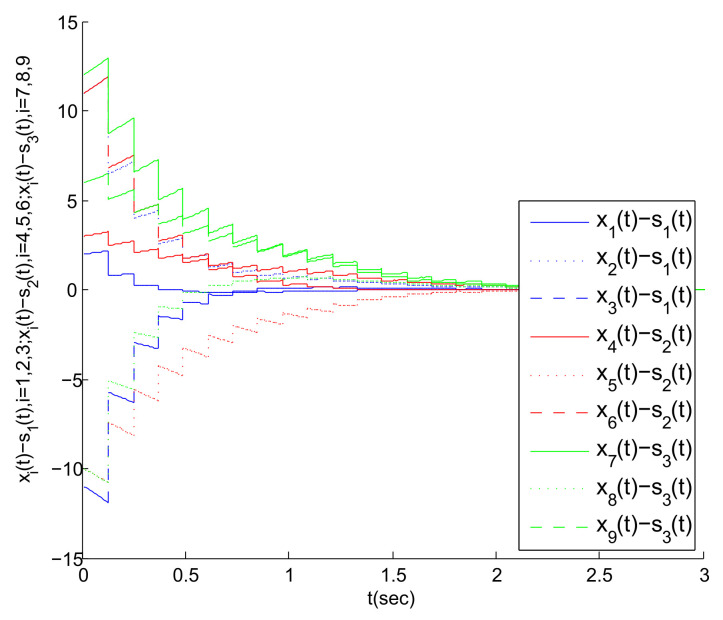
The state error trajectory for each agent in clusters.

We know that by adjusting the size of the impulsive interval during the simulation process and observing the impact of this operation on the speed of the multi-agent systems to achieve cluster mean square consensus, it can verify the dynamic relationship between the selection of parameter ϑ and the preset layout of the impulse time windows. We assume that there exists a parameter 1<ϑ<1.2 such that $Z_{k+1}^{l}-Z_{k}^{l}=0.12$ and $t_{k+1}-t_{k}=0.12$, and Figure 7 is obtained. By Figure 7, as described in Remark 5, it takes longer for SMASs to achieve cluster mean square consensus. Thus, the discussions in Remark 5 are reasonable.

**Figure 8:**
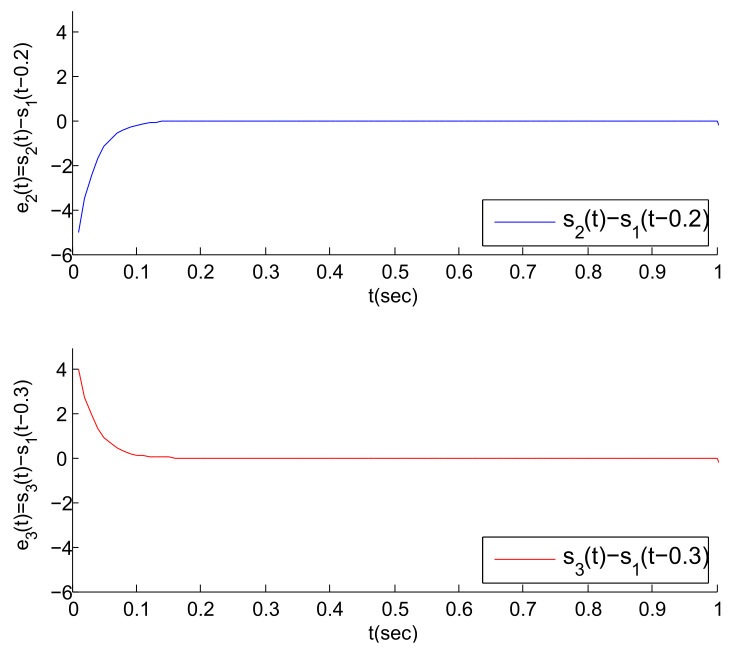
The error trajectories between $S_{2}\left(t\right)$, $S_{3}\left(t\right)$ and their virtual leaders’ states when $\rho_{2}=\rho_{3}=30$.

To verify the correctness of the theoretical analysis in Remark 6, we increase the value of the coupling strengths. That is, let $\rho_{2}=\rho_{3}=30$, and Figure 8 is obtained. According to Figure 8, it can be found that the two error trajectories in the figure can approximately converge to 0 at about 0.15. This convergence speed is obviously faster than that in Figure 6. Therefore, the obtained results in Remark 6 are correct.

## 6. Conclusions

Based on the discrete impulsive control strategy, this paper studies the cluster-delay mean square consensus problem of a class of SMASs with impulse time windows. According to the algebraic graph theory and Lyapunov stability theory, sufficient consensus criteria are given, and the obtained results are more general than the existing work. Moreover, according to the obtained conditions, the upper bound of the interval between the left endpoints of the two adjacent windows can be derived, which is conducive to the reasonable setting of the windows, so as to ensure that the cluster-delay consensus of SMASs in the mean square sense can be realized. Finally, a simulation example is designed to analyze and verify the feasibility of the relevant results. However, the research work in this paper still has some shortcomings. For instance, the dynamic model of each agent is homogeneous, and there are fewer objective factors considered in the system. Due to the wide application of heterogeneous MASs in practical applications, it is necessary to extend existing research work to heterogeneous MASs. In addition, considering the influence of factors such as time delay and switching topology in this paper is also a meaningful direction for work in the future.

## Data Availability

Not applicable.

## Abbreviations

The following abbreviations are used in this manuscript:

MASs	Multi-agent systems
SMASs	Stochastic multi-agent systems
ITM	Impulse time window
